# The expanding roles of Nr6a1 in development and evolution

**DOI:** 10.3389/fcell.2024.1357968

**Published:** 2024-02-19

**Authors:** Jingxuan Li, Pauline Mascarinas, Edwina McGlinn

**Affiliations:** Australian Regenerative Medicine Institute, Monash University, Clayton, VIC, Australia

**Keywords:** Nr6a1, GCNF, orphan nuclear receptor, axial elongation, *Oct4*, *Hox* genes, retinoic acid

## Abstract

The Nuclear Receptor (NR) family of transcriptional regulators possess the ability to sense signalling molecules and directly couple that to a transcriptional response. While this large class of proteins are united by sequence and structural homology, individual NR functional output varies greatly depending on their expression, ligand selectivity and DNA binding sequence specificity. Many NRs have remained somewhat enigmatic, with the absence of a defined ligand categorising them as orphan nuclear receptors. One example is Nuclear Receptor subfamily 6 group A member 1 (Nr6a1), an orphan nuclear receptor that has no close evolutionary homologs and thus is alone in subfamily 6. Nonetheless, Nr6a1 has emerged as an important player in the regulation of key pluripotency and developmental genes, as functionally critical for mid-gestational developmental progression and as a possible molecular target for driving evolutionary change in animal body plan. Here, we review the current knowledge on this enigmatic nuclear receptor and how it impacts development and evolution.

## Introduction

### Nuclear receptors: an overview

The Nuclear Receptor superfamily of transcriptional regulators are generally known to be intracellular receptors whose conformational change in response to ligand binding leads to a direct effect on transcription (for general overview see Frigo et al., 2021). NRs are found throughout the animal kingdom, ranging from 2 NRs found in the sponge *Amphimedon queenslandica* (Bridgham et al., 2010), 48/49 NRs found in human/mouse respectively and upwards to 73/74 in teleosts such as zebrafish and tilapia (Zhao et al., 2015). Phylogenetic analysis has divided the NR superfamily into 7 structurally distinct groups (NR0-NR6; Bridgham et al., 2010; Holzer et al., 2017) that can be broadly clustered into 3 branches: steroid hormone-related, thyroid hormone-related and retinoid X receptor-related. This complex diversification of NRs across animal lineages has provided insight into their possible ancestral functions and raising questions as to whether the ancestral NR was even ligand regulated (reviewed in Holzer et al., 2017).

Some well characterised NR ligands include thyroid hormone, steroid hormones such as estrogen, progesterone and glucocorticoids, as well as Vitamin A and Vitamin D derivatives. For the most part, these small lipophilic molecules freely diffuse across cell membranes, except for thyroid hormone that requires receptor-mediated transport. Once internalised, these well characterised examples act as high affinity ligands for their cognate receptor. However, it is also clear that many NRs bind various metabolites and lipids with low affinity, increasing the complexity of NR-ligand interactions.

With such a diverse and notable list of ligands, it is not surprising that NR activity is critical throughout the course of animal life, including early embryonic growth and patterning, developmental transitions and metamorphosis, reproduction, metabolism, and adult homeostasis (McKenna et al., 1999; Escriva et al., 2000; Robinson-Rechavi et al., 2003). Moreover, the dysregulation of NR signalling in many human pathological states including diabetes, multiple cancers, cardiovascular diseases, asthma, and neurologic syndromes (Ranhotra, 2013; Oyekan, 2011; Lonard and O'Malley, 2012; Kadmiel and Cidlowski, 2013; Mazaira et al., 2018) has led to intense interest in targeting NR function therapeutically. Indeed, various estimates suggest 15%–20% of currently available therapeutic drugs modulate NR function. However, only about half of the human NRs have known ligands, those with uncharacterised ligands being termed as orphan NRs. This review will focus on the orphan nuclear receptor Nuclear receptor subfamily 6 group A member 1 (Nr6a1), identified in 1994 and originally called Germ cell nuclear factor (GCNF), Retinoid receptor-related testis-associated receptor (RTR) or Neuronal cell nuclear factor according to the varied contexts in which the same factor was identified (Chen et al., 1994; Hirose et al., 1995; Bauer et al., 1997).

### Nr6a1: structural insight

**FIGURE 1:**
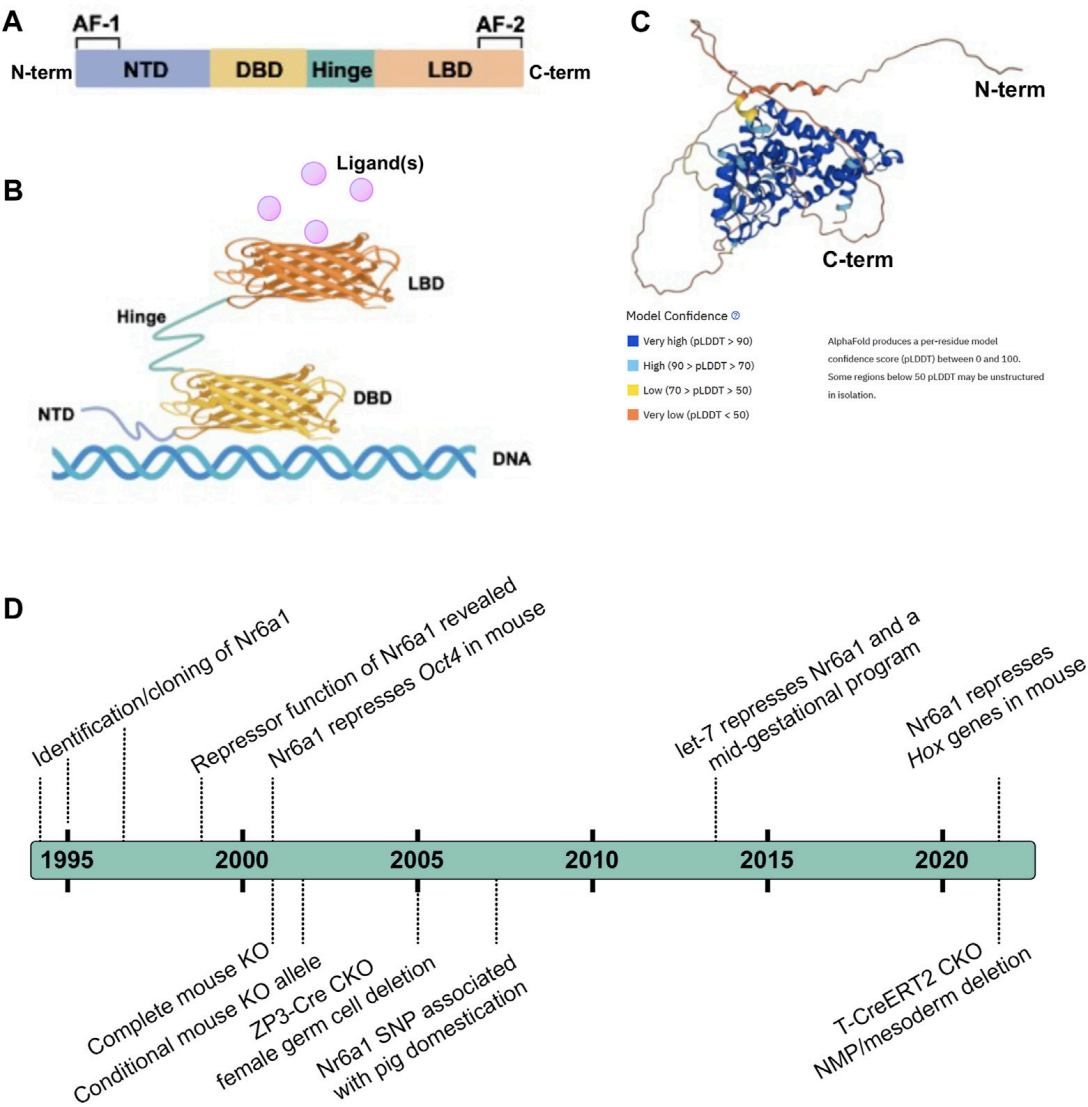
Nr6a1 Structural insight **(A,B)** Schematised structure of a canonical Nuclear Receptor. Nuclear receptors have a less conserved N-terminal domain (NTD) which harbours an activator function-1 (AF-1) region, a highly conserved DNA binding domain (DBD), a ligand-binding domain (LBD) that harbours an activator function-2 (AF2), and a hinge domain linking the DBD and LBD. **(C)** Predicted 3-Dimensional structure of human Nr6a1, generated by AlphaFold (Jumper et al., 2021; Varadi et al., 2022), model confidence indicated. **(D)** A timeline of key milestones in the identification and functional assessment of Nr6a1.

The consensus NR structure is composed of *i*) a poorly conserved N-terminal domain (NTD) in terms of length and sequence, that usually harbours an activator function-1 (AF-1) region that interacts with transcriptional coregulator proteins, *ii*) a highly conserved DNA binding domain (DBD), *iii*) a discrete ligand-binding domain (LBD) that not only interacts with ligand(s) but also recruits transcriptional coregulator proteins, and *iv*) a hinge region that connects the DBD and the LBD (Figure 1) (Escriva et al., 2000; Khorasanizadeh and Rastinejad, 2001; Bain et al., 2007; Gallastegui et al., 2015). Nr6a1 occupies subfamily group 6 alone and, despite its earlier naming as retinoid receptor-related testis-associated receptor, is more closely related to proteins of the steroid hormone branch (Papageorgiou et al., 2021). Compared with other NRs, the LBD region of Nr6a1 lacks an activator function-2 (AF-2) domain though the corresponding region can facilitate co-repressor recruitment and dimerization (Greschik et al., 1999; Zechel, 2005). Collective studies based largely on classical gel mobility shift assays have shown the DNA binding region of Nr6a1 binds with higher affinity as a homodimer than monomer, to a direct repeat with zero spacing (DR0) of the consensus sequence AGGTCA or an extended half site TCAGGTCA (Chen et al., 1994; Borgmeyer, 1997; Yan et al., 1997; Cooney et al., 1998; Schmitz et al., 1999). At least *in vitro*, Nr6a1 does not dimerize with the retinoid X receptor (Borgmeyer, 1997). Gu et al. (2005) suggested that endogenous Nr6a1 forms an even larger oligomeric complex called transiently retinoid-induced factor (TRIF) that requires DNA to assemble. Certainly, the half-site sequence was corroborated as enriched in Nr6a1-bound regions using chromatin immunoprecipitation of a Flag-HA-tagged Nr6a1 protein expressed in mesenchymal stem cells (Gurtan et al., 2013), and the exact nature of what higher order protein complex Nr6a1 forms *in vivo* still requires clarification.

### Dynamic *Nr6a1* expression

Initial characterisation of *Nr6a1* expression in adult mouse and human tissues revealed exponentially higher levels of *Nr6a1* in the testis compared to other organs, with low expression noted in ovary, kidney, and lung tissues (Chen et al., 1994). Cellular analysis by *in-situ* hybridization demonstrated *Nr6a1* was localised to the germ cells of the male testis and the female ovary (Chen et al., 1994). In male germ cells, high expression was noted during the final stage of round spermatid development with dramatic reduction during elongation of the spermatid cells, suggesting *Nr6a1* may act as a developmentally restricted post meiotic factor (Katz et al., 1997; Cooney et al., 1999; Yang et al., 2003). In developing oocytes however, *Nr6a1* expression was detected before the completion of meiosis but not in the primordial follicles, supporting an earlier role for Nr6a1 in this context during the initiation of oogenesis (Katz et al., 1997).

During mouse embryonic development, *Nr6a1* was detected as early as embryonic day (E) 6.5 in the ectoderm, with expression continuing throughout gastrulation stages in both anterior and posterior neuroepithelium as well nascent mesoderm emerging from the posterior primitive streak (Fuhrmann et al., 2001). At E8.5, expression appeared specific in the neural ectoderm and posterior growth zone (Chung et al., 2001), at least at the level of whole mount *in situ* hybridisation, with an absence of expression in the developing heart. Exploration of an E8.5 mouse single cell sequencing dataset (Pijuan-Sala et al., 2019) has confirmed widespread expression in cell types of all 3 germ layers, with high frequency across the caudolateral epiblast and bipotential neuromesodermal (NMP) progenitor populations (Chang et al., 2022), both key progenitor sources of the developing spinal cord and vertebral column (Henrique et al., 2015). By E9.5, expression remains strong in the anterior two-thirds of the embryo, including developing cranio-facial structures, limbs, neural tube and somites of the trunk, but is being visibly cleared from the posterior presomitic mesoderm and tailbud region (Chung et al., 2001; Chang et al., 2022). At E10.5, an overall reduction in Nr6a1 is observed, with cell-restricted expression in what appears to be trunk dorsal root ganglia and migrating cranial neural crest, though further characterisation is required. By E12.5, most *Nr6a1* expression is extinguished, highlighting a tightly controlled and temporally-restricted mode of transcript regulation.

**FIGURE 2:**
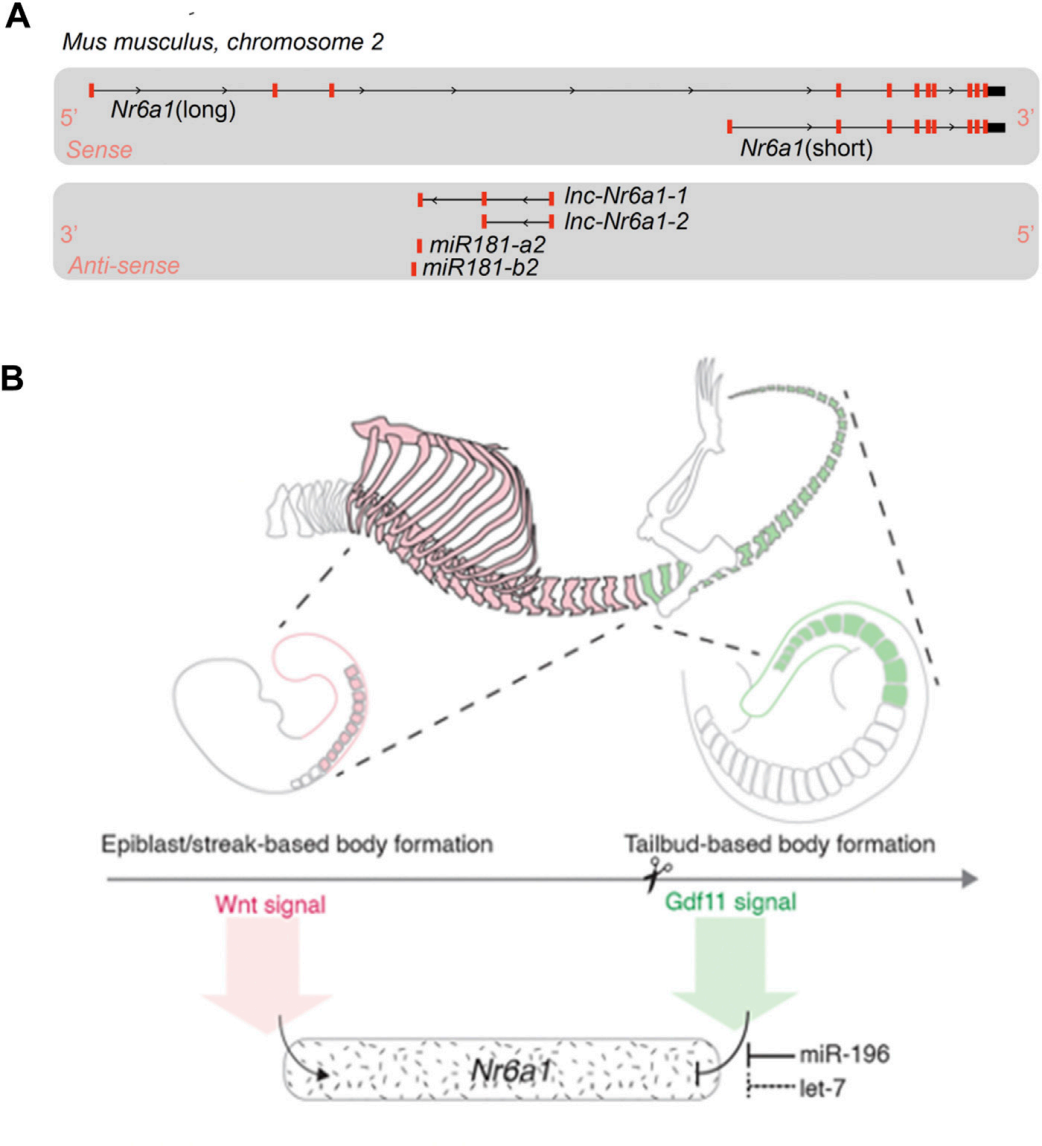
*Nr6a1* genomic structure and transcript regulation **(A)** The *Nr6a1* genomic locus of *Mus musculus.* Exons marked in red, not to scale. Multiple *Nr6a1* transcripts with coding potential have been identified on the sense strand, while both long non-coding antisense and micro-RNA encoding transcripts are produced from the opposite strand. **(B)** *Nr6a1* expression is defined by key developmental signals/regulators known to control axial elongation. *Nr6a1* expression increases in response to Wnt signalling, while the synergistic actions of Gdf11 and miR-196, and potentially let-7 expression, terminate *Nr6a1* expression at the trunk-to-tail transition.

It should be noted that both initial and more recent *in situ* expression characterisation utilised a riboprobe detecting the 3′-UTR region of *Nr6a1* transcript. With the vast wealth of transcript sequencing now available, it is clear the *Nr6a1* genomic locus produces a multitude of transcript isoforms which can impact protein coding potential (Figure 2A). On the sense strand, there are at least 2 major alternate transcripts with coding potential and many additional transcripts (not depicted) where coding potential is not defined and thus are likely non-coding RNAs (ncRNAs). Moreover, on the antisense strand, two partially overlapping long ncRNA transcripts (*lnc-Nr6a1-1* and *lnc-Nr6a1-2*) and a microRNA-encoding transcript (*mir-181-a2* and *mir-181-b2*) are produced. Recent *in vitro* analysis has shown that Tgf-β induced epithelial-to-mesenchymal transition rapidly upregulates all antisense transcripts, with *lnc-Nr6a1-1* and both pre-miRNAs being initially transcribed as a larger single unit (Polo-Generelo et al., 2022). At present, it is unclear if and how Nr6a1 protein functionality is controlled at the level of alternative transcript expression, nor whether there is coordinated regulation of sense and antisense transcripts *in vivo*—either positive or inverse correlation—as is often observed for sense-antisense pairs.

### Regulatory mechanisms controlling *Nr6a1* expression

The timely induction and termination of *Nr6a1* expression during development and differentiation is essential. The first clues as to regulatory factors capable of inducing *Nr6a1* expression came following its identification as an early response target gene in embryonic carcinoma cells that were induced by retinoic acid (RA) towards a neural cell fate (Bauer et al., 1997; Heinzer et al., 1998). This finding was subsequently confirmed in both mouse (Sato et al., 2006) and human (Wang et al., 2016) embryonic stem cells treated with RA, and *in vivo* following exogenous application of RA to the *Xenopus* embryo (Barreto et al., 2003). Interestingly, some level of negative feedback exists between Nr6a1 and the key RA biosynthesis enzyme *Aldh1a2*, whereby complete loss of Nr6a1 *in vivo* results in a spatially discrete upregulation of *Aldh1a2* in the tailbud (Chung et al., 2001), and conversely, *Aldh1a2* is one of the most downregulated transcripts in the tailbud following *in vivo* Nr6a1 overexpression (Chang et al., 2022). Additional inducers of high level *Nr6a1* expression have been identified *in vitro*, including the Fibroblast Growth Factor and the Wnt signalling pathways (Chang et al., 2022). During ESC differentiation to the bipotential NMP progenitor, the addition of FGF2 (inducing ESC to epiblast-like transition) resulted in an approx. 80-fold increase of *Nr6a1*, while subsequent addition of the Wnt pathway agonist CHIR99021 (inducing epiblast-like to NMP transition) led to a further increase to 175-fold relative to ESCs. *In vivo* spatio-temporal context of the relative contributions to induction remains to be delineated, however Wnt/Fgf-dependency correlates well with the strong *in vivo* posterior expression of *Nr6a1* between E7.5-E8.5.

Slightly later in development, both *in situ* hybridisation and single cell RNAseq analysis have demonstrated a sharp clearance of *Nr6a1* from the wildtype posterior growth zone at a key developmental transition known as the trunk-to-tail transition (approximately E9.5 in mouse). This transition marks the end of primary body elongation and is temporally regulated by both Gdf11 signalling (McPherron et al., 1999; Jurberg et al., 2013) and the miR-196 family of microRNAs (Wong et al., 2015), likely across vertebrate species (He et al., 2011; Matsubara et al., 2017). Our group has shown that addition of Gdf11 to *in vitro*-derived NMP cells almost completely abolishes *Nr6a1* expression, back to the low levels seen in ESCs (Chang et al., 2022). *In vivo*, Gdf11 and miR-196 were shown to have individual and an additive role in the timely clearance of *Nr6a1* at this site, with the clearance of Nr6a1 functionally mediating, at least in part, the role of Gdf11 at this critical transition point. Interestingly, the let-7 family of microRNAs are also predicted to extensively target both mouse and human *Nr6a1* 3′-UTRs (McGeary et al., 2019) and thus could be predicted to also participate in the timely clearance of *Nr6a1*. Let-7 has been experimentally validated to suppress *Nr6a1* as part of a broad “mid-gestational” genetic signature in murine mesenchymal stem cells (Gurtan et al., 2013), and genetic reduction of let-7 paralogs in the mouse increased tail vertebral number by 5 elements (Robinton et al., 2019). Whether this latter phenotype is again in part due to a de-repression of Nr6a1 is not known, though perhaps unlikely given that transgenic overexpression of *Nr6a1* in the posterior tailbud revealed the presence of Nr6a1 is detrimental to tail vertebrae morphology (Chang et al., 2022). Whether let-7 may act redundantly with Gdf11 and miR-196 at slightly more rostral locations however is possible and would require complex mouse genetics to dissect. In summary, *Nr6a1* expression is tightly controlled during formation of the embryo by key developmental signals and post-transcriptional regulatory mechanisms, several of which are considered as regulators of developmental timing, prompting a detailed functional dissection in this context.

### *Nr6a1* is essential for embryonic survival

Complete genetic deletion of *Nr6a1* in the mouse has revealed its indispensable role during mid-gestation development and in embryonic survival (Chung et al., 2001). The very early stages of *Nr6a1* ^*−/−*^ embryogenesis did not appear to be overtly compromised however, by E8.5, *Nr6a1* ^*−/−*^ embryos began to exhibit clear morphological defects including an open neural tube and a disorganisation of the primitive streak and posterior tissues that exacerbated over time. The allantois was larger than WT with defects in chorion attachment, while tissue of the posterior embryo proper began extending abnormally and were positioned outside of the yolk sac. By E9.5, *Nr6a1* ^*−/−*^ embryos had failed to rotate the posterior half of the main body axis as is normally observed by this stage in WT embryos and exhibited additional posterior alterations in hindgut and ventral body wall development. Somitogenesis was severely compromised, and overall embryo growth was stunted, though some regionalised expansion of the very anterior and posterior tissue was still observed at this stage. The latest time point where viable *Nr6a1* ^*−/−*^ embryos were recovered was E10.5, albeit at ratios lower than Mendelian expectations, with only resorbing *Nr6a1* ^*−/−*^ embryos observed at E11.5. The cause of lethality has not been precisely characterised but is likely due to altered chorioallantoic attachment and/or pericardial distention. The aetiology of this latter defect is not immediately obvious since *Nr6a1* expression appears to be largely absent from the heart at E8.5-9.5, but whether Nr6a1 functions within very early mesodermal heart field(s) remains possible. An important molecular target of Nr6a1, the pluripotency factor *Oct4*/*Pou5f1*, was found to be ectopically expressed across much of the somatic tissue in E8.5 Nr6a1 null embryos (Fuhrmann et al., 2001), a time when this gene normally becomes highly restricted to the germline. These comprehensive studies, and the subsequent ubiquitous deletion of *Nr6a1* DBD using Cre/LoxP technology that phenocopied early Nr6a1 null results (Lan et al., 2002), have provided the broad strokes for understanding Nr6a1 requirements during early development. However, the catastrophic defects resulting from global Nr6a1 deletion limited the ability to characterise later embryonic or adult requirements, or tissue-specific functions, of this important regulator.

### Nr6a1 is essential for male and female germ cell development

Using the original Nr6a1 null allele, it was shown that initial segregation of the germline cell lineage (primordial germ cells; PCGs) during early embryogenesis, and early PGC migration, do not require Nr6a1 function (Sabour et al., 2014). As Nr6a1 null embryos die soon after these stages, *ex vivo* knockdown of Nr6a1 (Nra61-KD) within testes-derived germline stem cells was performed, with transduced cells re-introduced into germ-cell depleted seminiferous tubules. Both control and Nr6a1-KD cells were able to re-colonise this environment, but by 3 months, Nr6a1-KD cells never produced functional sperm as compared to controls (Sabour et al., 2014). To dissect the role of Nr6a1 within maturing oocytes of an adult female mouse, a Zp3-Cre conditional knock-out model was employed (Lan et al., 2003). Phenotypically, homozygous conditional deletion did not affect germ cell number but led to reduced fertility owing to an extended diestrus of the estrus cycle, abnormal steroidogenesis, and double-oocyte follicles. The molecular aetiology of these largely non-cell autonomous consequences stemmed from the loss of repression specifically at diestrus of *Bmp-15* and *Gdf-9*, two members of the TGF-b family of secreted ligands known to be critical for female reproduction (Carabatsos et al., 1998; Erickson and Shimasaki, 2001; Zhao et al., 2007). This led to a reduction of follicle stimulating hormone (FSH) and somatic cell-produced steroid hormones specifically in diestrus, underpinning the observed phenotypic consequences. In contrast, a separate conditional approach deleting the LBD of Nr6a1 from E10.5 using a ubiquitous by temporally controlled Cre deleter line observed a surprising lack of effect on the initiation of meiosis or early oogenesis (Okumura et al., 2013). A subsequent *in vivo* chimeric approach, using Nr6a1-null ESCs injected into a WT blastocyst stage embryo, revealed a reduced contribution to the germline in the absence of Nr6a1 activity (Sabour et al., 2014). These chimeric gonads were then implanted under the kidney capsule to develop further, with only degenerated oocytes present at 4 weeks post-transplant in Nr6a1-null chimeric gonads compared to normal oocyte development observed in the WT-chimeric controls.

### Nr6a1 has a regionally-critical role in elongation of the main body axis

The vertebral column and spinal cord arise from progenitors of the posterior growth zone, with tissue being sequentially constructed over a series of days in an anterior-to-posterior (A-P; head-to-tail) direction. The abrupt termination of *Nr6a1* expression from across the wildtype E9.5 posterior growth zone (Chung et al., 2001), supported by single cell RNAseq analysis of *in vivo* NMPs (Gouti et al., 2017), suggested that Nr6a1 function may be regionally-restricted during vertebrate axial elongation. To test this, conditional deletion of Nr6a1 using a tamoxifen-inducible Cre deleter line active in axial progenitors and early mesoderm (*TCre* ^*ERT2*^) was employed, circumventing the early embryonic lethality observed in Nr6a1 null embryos and allowing analysis of skeletal alterations in late stage embryos. Compared to the WT axial formulae of 7 cervical (C), 13 thoracic (T), 6 lumbar (L), 4 sacral (S) and 30-31 tail vertebrae, conditional knockout (CKO) of Nr6a1 activity from E7.5 resulted in a dose-dependent reduction in the number of trunk vertebral elements with increasing vertebra dysmorphology and rib fusions (Chang et al., 2022). CKO of one Nr6a1 allele reduced thoracic number by 2, while CKO of both Nr6a1 alleles resulted in 4 fewer thoracic elements. In each of these mutant scenarios, total vertebral number was reduced by 1 and 3 elements respectively, confirming the requirement for Nr6a1 in maintaining axial elongation. In parallel, while the entire lumbar region of *TCre* ^*ERT2*^; *Nr6a1* ^*flx/flx*^ embryos was not altered in terms of segment number, vertebral identity was transformed almost whole-sale to that of sacral elements based on characteristic lateral process morphology and fusion. This latter phenotype was particularly interesting since the developing hindlimbs, which normally align and ultimately articulate with sacral elements, were positioned normally in *TCre* ^*ERT2*^; *Nr6a1* ^*flx/flx*^ embryos, supporting a disassociation of the patterning events between hindlimb-forming lateral plate mesoderm and vertebral column-forming paraxial mesoderm. Finally, while the majority of thoraco-lumbar-sacral elements in *TCre* ^*ERT2*^; *Nr6a1* ^*flx/flx*^ embryos were highly dysmorphic, all post-sacral vertebral elements reverted back to WT morphology. This phenotype could be traced back to segmentation stages (E10.5) where a striking switch back to normal somite morphology could be observed immediately after the last sacral-forming somite (Chang et al., 2022).

In contrast to the above conditional loss-of-function scenario, Nr6a1 gain-of-function in the mouse posterior growth zone using a transient transgenic approach yielded almost mirror-image phenotypic alterations (Chang et al., 2022). These included an increased number of phenotypically normal thoraco-lumbar vertebrae by up to 5 elements, and a sharp switch this time to highly dysmorphic post-sacral elements and tail truncation. Together, these results not only delineated the critical requirement for Nr6a1 in axial elongation, but they also revealed quite unique phenotypes that will drive further research into lineage-specific patterning requirements and into axially-restricted gene regulatory networks that impact the seemingly uniform process of segmentation.

### Molecular mechanisms and targets of Nr6a1

Nr6a1 has been characterised as a repressor of gene expression (Cooney et al., 1998; Yan and Jetten, 2000), with all evidence to date supporting this as its sole regulatory function. Early work revealed many examples of Nr6a1-dependent repression in germ cells that relied on the presence of a DR0 site, including protamine 1 and 2, mGDPH and ELP in male germ cells (Yan et al., 1997; Hummelke et al., 1998; Yang et al., 2003) and BMP-15 and Gdf-9 in female germ cells (Carabatsos et al., 1998; Erickson and Shimasaki, 2001; Zhao et al., 2007).

**FIGURE 3:**
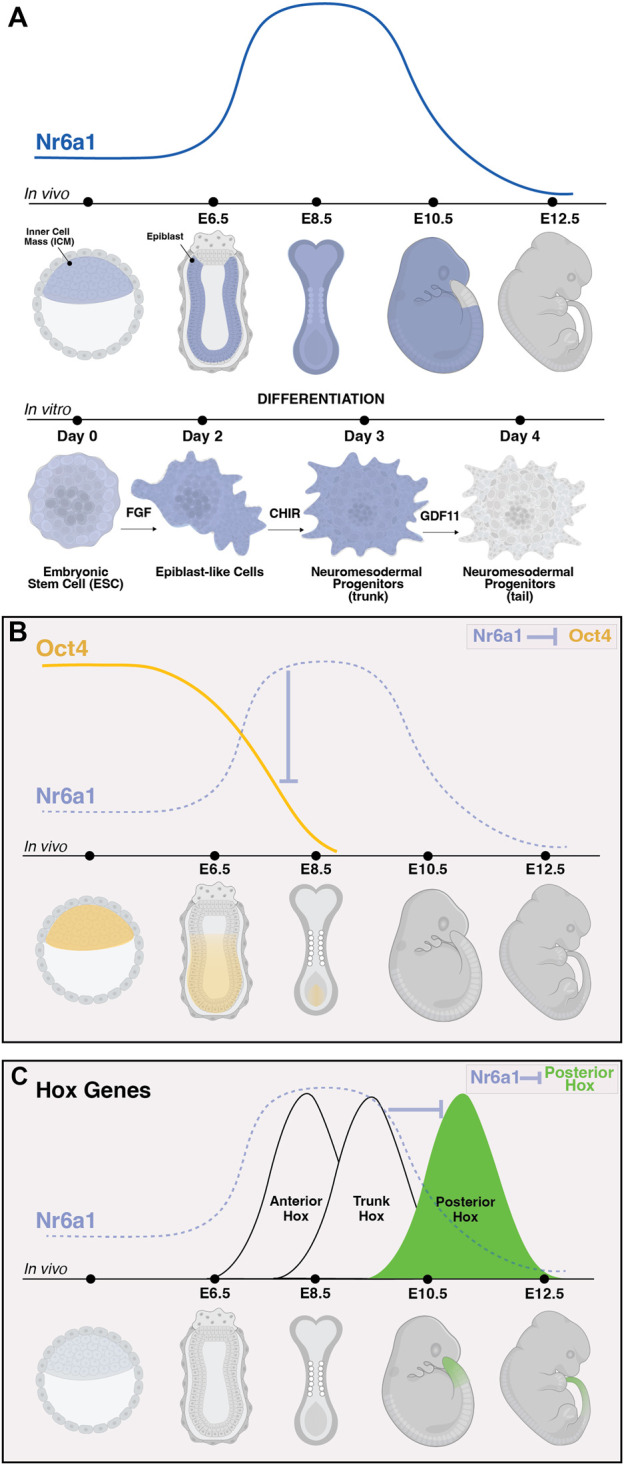
The dynamic expression of *Nr6a1* controls key developmental genes and transitions. The expression of Nr6a1 over time *in vivo* and during *in vitro* differentiation **(A)**. *In vivo*, *Nr6a1* expression is detected within the inner cell mass (ICM) at very early stage of mouse development, broadly within the epiblast (epi) at embryonic day (E)6.5, across most tissues and germ layers at E8.5, with a gradual clearing of expression beginning with the posterior tail bud from E9.5 and expression largely cleared from the embryo by E12.5. A similar expression dynamic is seen during *in vitro* ESC-to-NMP differentiation: *Nr6a1* expression within ESCs increases following exposure to Fgf2, and further increases following activation of Wnt signalling (CHIR). The transition *in vitro* from a trunk NMP to a tail NMP following exposure to Gdf11 downregulates *Nr6a1* to low/basal levels. The dynamic *in vivo* expression of *Nr6a1* overlaid with key target genes *Oct4* and posterior *Hox* genes **(B,C)**. The rise of Nr6a1 within the epiblast directly repress *Oct4* levels, leading to broadly complementary patterns of expression between early and mid-gestation **(B)**. Conversely, the rise of Nr6a1 prevents precocious expression of posterior Hox genes, leading to broadly complementary patterns of expression between mid and late-gestation **(C)**. Whether posterior *Hox* repression by Nr6a1 is via direct mechanisms is currently unclear. Images created in Biorender.

Certainly, one of the most notable target gene networks directly influenced by Nr6a1 activity is that of the pluripotency network. High expression levels of *Pou5f1*/*Oct4* are part of a core network maintaining pluripotency within the *in vivo* mouse blastocyst and within *in vitro* blastocyst-derived ESCs. As differentiation proceeds in either system, the decrease and eventual termination of *Oct4* expression was shown to inversely correlate with a rise in *Nr6a1* expression levels (Figures 3A,B) (Fuhrmann et al., 2001; Gu et al., 2005), consistent with Nr6a1’s known repressive mode of action. A DR0 sequence was identified immediately upstream of the mouse *Oct4* transcription start site (Fuhrmann et al., 2001), and subsequently across many vertebrate species (Wang et al., 2016), with direct binding to this site confirmed in mouse embryonal cells, mouse ESCs and human ESCs. In these various contexts, the Nr6a1 protein has been shown to directly interact with SMRT and N-Cor corepressor proteins in mouse embryonal cells (Fuhrmann et al., 2001), DNMT3b methyltransferase in human ESCs (Sato et al., 2006), and both Dnmt3A and methyl-CpG binding domain (MBD) proteins MBD-2 and MBD-3 in mouse ESCs (Gu et al., 2011), supporting histone deacetylation and *de novo* DNA methylation as ultimate mechanisms by which Nr6a1 reduces *Oct4* target gene expression. Like*Oct4*, a second key pluripotency gene *Nanog* is directly repressed by Nr6a1, with indirect repressive consequences on the larger network including *Sox2*, *Stella* and *Fgf4* (Gu et al., 2005).

During later tailbud stages in the mouse, global expression changes elicited by *in vivo* ectopic Nr6a1 activity revealed the maintenance of a core trunk gene regulatory network (Chang et al., 2022) longer than wildtype, consistent with the Nr6a1-dependent “mid-gestation program” identified in mesenchymal stem cells (Gurtan et al., 2013). Of particular importance was the identification that Nr6a1 activity significantly impacts the timing of gene activation across all 4 *Hox* clusters, central regulators of body plan formation across bilateria (reviewed in Hubert and Wellik, 2023). Ectopic expression of Nr6a1 stalled progression of *Hox* gene activation at a trunk (Hox5-9) code with a concomitant downregulation (or likely delayed activation) of posterior/terminal (*Hox11*-*13*) genes. Conversely, loss of Nr6a1 activity, both *in vitro* and *in vivo*, led to a speeding up of *Hox* cluster progression and precocious activation of posterior/terminal *Hox* genes (Figure 3C) (Chang et al., 2022). In parallel, Nr6a1 was shown to impact the balance of neural vs. Mesodermal gene signatures in the tailbud. Further work is needed to understand the direct vs. Indirect regulatory nature of each of these interactions and whether changes in histone marks and DNA methylation are involved.

### Nr6a1 and the evolution of animal body plan

The body plan and ensuing axial formulae of a given vertebrate species is remarkably robust, particularly for isogenic mouse strains such as C57Bl6 that are used in many genetic studies. In contrast, the diversity of body plans across the vertebrate species can be extreme, and the molecular mechanisms driving such changes are of intense interest yet still largely unknown. As the precise expression level of Nr6a1 has now been shown to control total vertebral number in the mouse, both positively and negatively, this work raises the possibility that Nr6a1 may be a molecular target for evolutionary change. In support of this view, numerous studies have identified a genetic association between Nr6a1 and the increase in vertebral count of domesticated animals, a trait possibly selected for in the livestock industry due to its advantage in boosting meat yield. In 2005, Mikawa et al. (2005) conducted a quantitative trait loci (QTL) analysis comparing domesticated pigs and wild boar populations, pinpointing regions on *Sus scrofa* chromosomes 1 and 7 as associated with an increase of more than two presacral vertebrae. Subsequent fine mapping of the chromosome 1 loci identified a C>T single nucleotide polymorphism (SNP) at nucleotide 748 of *Nr6a1* which segregated with the phenotype (Mikawa et al., 2007). This SNP led to a non-conservative amino acid substitution of proline to leucine at amino acid 192 within the protein’s hinge region which, using a yeast-two-hybrid assay, led to enhanced binding between Nr6a1 and the corepressors NCOR1 and RAP80. The predicted molecular gain-of-function aligns well with phenotypic outcomes of *in vivo* Nr6a1 gain-of-function studies in increasing thoraco-lumbar number (Chang et al., 2022). Subsequent studies by Zhang et al. (2019) have discovered an A>C SNP within exon 8 of Nr6a1 that potentially influences the number of lumbar vertebrae in sheep. Similarly, Fang et al. (2019) identified a 13 bp deletion within intron 1 of NR6A1 in various donkey breeds, linking it to body size attributes such as height and length. The exact molecular consequences of these later polymorphisms are still to be delineated but support the possibility that subtle changes in Nr6a1 regulation and/or function may have been an important event in the evolution of intra-species variation. In this light, further analysis of Nr6a1 sequence and function in vertebrate animals with extreme body plans, such as the snake, would be of major interest.

## Conclusion

Nr6a1 is a critical developmental regulator with emerging roles in disease. It’s connection to key signalling pathways (RA, Wnt, and Fgf) and its ability to repress fundamental developmental molecules (*Oct4*, *Nanog*, posterior *Hox* genes) have been characterised in disparate contexts and a clearer consensus of the similarities and difference in how Nr6a1 functions across time and space is needed. What can be speculated however given the notable list of target genes this protein represses, and the quantitative manner in which Nr6a1 levels affect these targets (both positively and negatively), is that Nr6a1 could be utilised to guide positional identity and/or cell identity in 3D *in vitro* cell-based models of development either in its native form or as an engineered transcription factor. For example, manipulating the precise level of Nr6a1 is likely to “speed up” or “slow down” the *Hox* clock in 3D models of axial elongation such as gastruloids (Beccari et al., 2018) or somitoids (Sanaki-Matsumiya et al., 2022; Yamanaka et al., 2023). Along similar lines, the current inability of many cellular or organoid platforms to transition to a mature state, the often-lengthy (thus costly) protocols required, and the exhaustion of progenitor pools may all be enhanced by direct manipulation of positional identity via Nr6a1. Alternatively, Nr6a1 expression may provide a robust exit from pluripotency in blastoid models (Rivron et al., 2018; Liu et al., 2021; Yu et al., 2021) of early embryogenesis.

In future research, the dynamic transcriptional output of the genomic loci encompassing Nr6a1, Nr6a1 antisense transcripts and the two miR-181 microRNAs requires careful *in vivo* dissection, first to understand any co-regulation or anti-regulation that may shape Nr6a1’s spatio-temporal functional output and second, to determine if antisense transcripts have independent functional roles of as has been shown *in vitro* (Polo-Generelo et al., 2022). Once expressed, how is Nr6a1 protein subcellular localisation controlled and what, if any, ligand(s) trigger a downstream transcriptional response? What is the full complement of direct genomics targets of Nr6a1 *in vivo*, particularly in more recent areas of focus such as axial elongation, and how is lineage-restricted target gene regulation achieved? What higher order protein complexes is Nr6a1 guiding to the chromatin *in vivo* and is this lineage- or cell-restricted? And finally, how have the array of Nr6a1 SNPs identified across vertebrate species altered its molecular function and were these changes an important driver of phenotypic change across evolution.
